# A Sulfated-Polysaccharide Fraction from Seaweed *Gracilaria birdiae* Prevents Naproxen-Induced Gastrointestinal Damage in Rats

**DOI:** 10.3390/md10122618

**Published:** 2012-11-22

**Authors:** Renan O. Silva, Ana Paula M. Santana, Nathalia S. Carvalho, Talita S. Bezerra, Camila B. Oliveira, Samara R. B. Damasceno, Luciano S. Chaves, Ana Lúcia P. Freitas, Pedro M. G. Soares, Marcellus H. L. P. Souza, André Luiz R. Barbosa, Jand-Venes R. Medeiros

**Affiliations:** 1 LAFFEX—Laboratory of Experimental Physiopharmacology, Biotechnology and Biodiversity Center Research (BIOTEC), Federal University of Piauí-CMRV, Parnaíba 64202-020, PI, Brazil; Email: renan.oliveira25@yahoo.com.br (R.O.S.); natyfallen@hotmail.com (N.S.C.); thalita.bezerra@hotmail.com (T.S.B.); millaufpi@hotmail.com (C.B.O.); samarinharodrigues23@hotmail.com (S.R.B.D.); andreluiz@ufpi.edu.br (A.L.R.B.); 2 LAFICA—Laboratory of Pharmacology of Inflammation and Cancer, Department of Physiology and Pharmacology, Federal University of Ceará, Fortaleza 60430-270, CE, Brazil; Email: apmacedo1@hotmail.com (A.P.M.S.); pedrogsoares@ufc.br (P.M.G.S.); souzamar@ufc.br (M.H.L.P.S.); 3 Laboratory of Proteins and Carbohydrates of Marine Algae, Department of Biochemistry and Molecular Biology, Federal University of Ceará, Fortaleza, CE 60455-760, Brazil; Email: lucianoscsep@hotmail.com (L.S.C.); pfreitas@ufc.br (A.L.P.F.)

**Keywords:** sulfated polysaccharide, gastrointestinal damage, naproxen, antioxidant activity

## Abstract

Red seaweeds synthesize a great variety of sulfated galactans. Sulfated polysaccharides (PLSs) from seaweed are comprised of substances with pharmaceutical and biomedical potential. The aim of the present study was to evaluate the protective effect of the PLS fraction extracted from the seaweed *Gracilaria birdiae* in rats with naproxen-induced gastrointestinal damage. Male Wistar rats were pretreated with 0.5% carboxymethylcellulose (control group—vehicle) or PLS (10, 30, and 90 mg/kg, *p.o.*) twice daily (at 09:00 and 21:00) for 2 days. After 1 h, naproxen (80 mg/kg, *p.o.*) was administered. The rats were killed on day two, 4 h after naproxen treatment. The stomachs were promptly excised, opened along the greater curvature, and measured using digital calipers. Furthermore, the guts of the animals were removed, and a 5-cm portion of the small intestine (jejunum and ileum) was used for the evaluation of macroscopic scores. Samples of the stomach and the small intestine were used for histological evaluation, morphometric analysis and in assays for glutathione (GSH) levels, malonyldialdehyde (MDA) concentration, and myeloperoxidase (MPO) activity. PLS treatment reduced the macroscopic and microscopic naproxen-induced gastrointestinal damage in a dose-dependent manner. Our results suggest that the PLS fraction has a protective effect against gastrointestinal damage through mechanisms that involve the inhibition of inflammatory cell infiltration and lipid peroxidation.

## 1. Introduction

In recent years, marine resources have attracted attention as a source of bioactive compounds for the development of new drugs and healthy foods [1]. In particular, seaweeds are a very important and commercially valuable resource for the food industry; they also serve as soil conditioners and are used in traditional medicine because of their perceived health benefits [2,3]. In addition, sulfated polysaccharides (PLSs) from marine algae are known to exhibit many biological and physiological activities, including anticoagulant, antiviral, antitumor, anti-inflammatory, and antioxidant effects [4,5,6,7].

Red seaweeds synthesize a great variety of sulfated galactans, which are the major components of the extracellular matrix. Red seaweed galactans are of great commercial importance and are used widely in the food industry as gelling and thickening agents, due to their rheological properties [3]. These PLSs are primarily classified as agarans and carrageenans. In particular, galactans with 4-linked α-galactose residues of the l-series are termed agarans and those of the d-series are termed carrageenans [8]. Polysaccharides from the *Gracilaria* genus are composed mainly of the alternating 3-linked-β-d-galactopyranose unit (Gal) and the 4-linked-3,6-anhydro-α-l-galactopyranose unit (AnGal). The Gal unit can be substituted by either methyl or sulfate ester groups [9].

PLSs comprise a complex group of macromolecules with a wide range of important biological properties. The PLSs in seaweeds contain substances with great pharmaceutical and biomedical potential [10,11,12]. Recent studies have shown that PLSs extracted from *Gracilaria birdiae *demonstrated marked antioxidant and anti-inflammatory activities [11]. However, few studies have correlated the potential of PLSs from seaweeds to the gastrointestinal tract damages associated with the use of non-steroidal anti-inflammatory drugs (NSAIDs).

NSAIDs are often recommended clinically because of their anti-inflammatory, anti-pyretic, and analgesic properties. However, the chronic use of these drugs is limited as a result of their capacity to cause damage to the gastrointestinal tract, such as erosion, ulceration, perforation, and hemorrhage [13,14]. Naproxen is a non-selective NSAID that is widely prescribed for chronic treatments such as that for arthritis; it is also one of the most likely drugs in this class to induce gastrointestinal damage [15,16,17]. The production of oxygen free radicals and lipid peroxidation play important roles in the naproxen-induced gastric antral ulceration [18,19].

Thus, the aim of the present study was to investigate the protective effect of the PLS fraction extracted from *G. birdiae* on naproxen-induced gastrointestinal damage in rats. 

## 2. Results and Discussion

The polysaccharide fraction isolated from the red alga *G. birdiae* was previously identified [11,20]. This galactan is an agar-type polysaccharide composed mainly of β-d-galactopyranose linked to 3,6-anhydro-α-l-galactose with low methyl substituted groups. The structure is formed by →4-3,6-anhydro-α-l-gal*p *(1→3) β-d-gal*p* 1→ segments, with the possibility of an α-l-gal*p* unit substituted at the 6-position by a sulfate ester [20]. In addition, the molar mass distribution was found to be within 2.6 × 10^6^ and 3.75 × 10^5^ g/mol, while the soluble carbohydrate, protein, and sulfate contents were 85.5%, 2.5%, and 8.4%, respectively [11,20]. 

**Figure 1:**
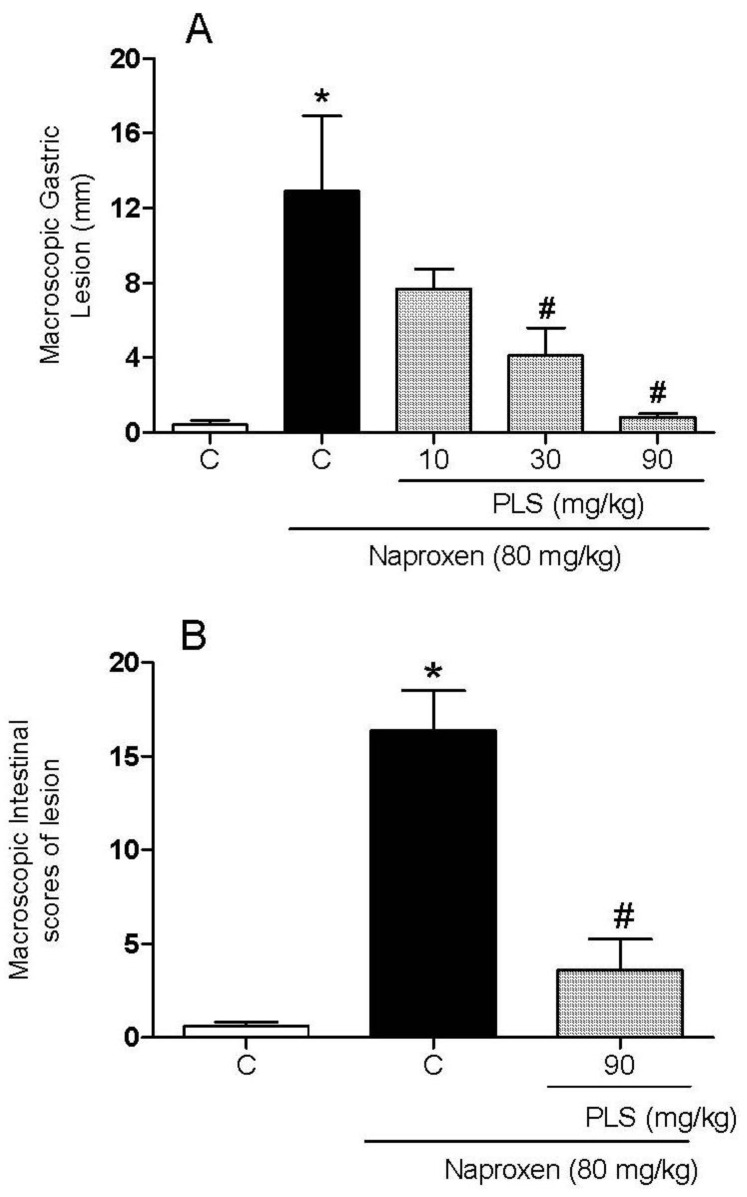
The sulfated-polysaccharide (PLS) fraction extracted from *Gracilaria birdiae* reduces naproxen-induced gastric (**A**) and intestinal damage (**B**). Rats were treated by gavage with either carboxymethylcellulose (C: control) or PLS (10, 30, and 90 mg/kg) twice daily (at 09:00 hours and 21:00 hours) for two days. After 1 h, naproxen (80 mg/kg) was administered by gavage. The results are expressed as the mean ± SEM of 5–7 animals per group. * *p *< 0.05 *vs. *carboxymethylcellulose group; # *p *< 0.05 *vs. *naproxen group (ANOVA and Newman-Keuls test).

NSAIDs, such as naproxen, are the most widely used pharmacological agents for the treatment of pain and inflammation [21]. However, 15%–30% of the patients who receive these drugs develop gastrointestinal ulcers [22]. The gastrointestinal protective effect of PLS extracted from *G. birdiae* was evaluated using a rat model of naproxen-induced gastrointestinal damage. In the present study, we confirmed that treatment of the animals with naproxen for two days led to the formation of severe macroscopic gastric and intestinal lesions (12.9 ± 4.0 mm and 16.4 ± 2.0 lesion scores, respectively). Moreover, PLS treatment was found to reduce the macroscopic and microscopic naproxen-induced gastrointestinal damage. Figure 1 shows that PLS prevented naproxen-induced gastropathy in a dose-dependent manner, reaching its maximal effect at a dose of 90 mg/kg, with 93% and 78% lesion inhibition in the stomach and small intestine (jejunum and ileum), respectively.

Gastric ulceration due to the ingestion of NSAIDs appears to be associated with a reduction in gastric blood flow and an increase in leukocyte adherence within the gastric microcirculation, secondary to a reduction in prostaglandin synthesis [23]. Several research groups have demonstrated that serious NSAID-associated gastrointestinal complications develop not only in the upper, but also in the lower gastrointestinal tract, including the small intestine; recently, this problem has become a topic of great interest to gastroenterologists [24]. Therefore, determining the mechanisms of NSAID-induced mucosal injury in the small intestine and identifying substances that effectively protect the gastrointestinal mucosal against these aggressors is of great importance.

The gastroprotective effect of 90 mg/kg PLS was confirmed by histological analysis (Table 1). Microscopic analysis revealed that naproxen increased edema, epithelial cell loss, and inflammatory cell infiltration, but did not cause hemorrhagic damage. On the other hand, pretreatment with PLS significantly decreased the infiltration of inflammatory cells, the formation of edema and the loss of epithelial cells induced by naproxen (Table 1). Thus, the analysis of both the macro- and microscopic findings revealed an excellent correlation, confirming the efficacy of the compound.

**Table 1:** Effect of the sulfated-polysaccharide fraction (PLS, 90 mg/kg) extracted from *Gracilaria birdiae* on naproxen-induced microscopic gastric damage.

Experimental group (*n* = 5)	Hemorrhagic damage (score, 0–4)	Edema (score, 0–4)	Epithelial cell loss (score, 0–3)	Inflammatory cells (score, 0–3)	Total (score, 0–14)
**Control**	0	0 (0–1)	0	0 (0–1)	0 (0–3)
**Naproxen**	0	3 (1–3) ^a^	3 (3–3) ^a^	2 (2–3) ^a^	7 (5–9) ^a^
**Naproxen + PLS (90 mg/kg)**	0	0 (0–1) ^b^	0 (0–2) ^b^	0 (0–1) ^b^	0 (0–3) ^b^

Data shown are the median values with the minimum and maximum scores given in parentheses. The Kruskal-Wallis nonparametric test followed by Dunn’s test were used for multiple comparisons of histological analyses. ^a^ *p* < 0.05 *vs. *control group (carboxymethylcellulose); ^b^ *p *< 0.05 *vs. *naproxen group.

**Figure 2:**
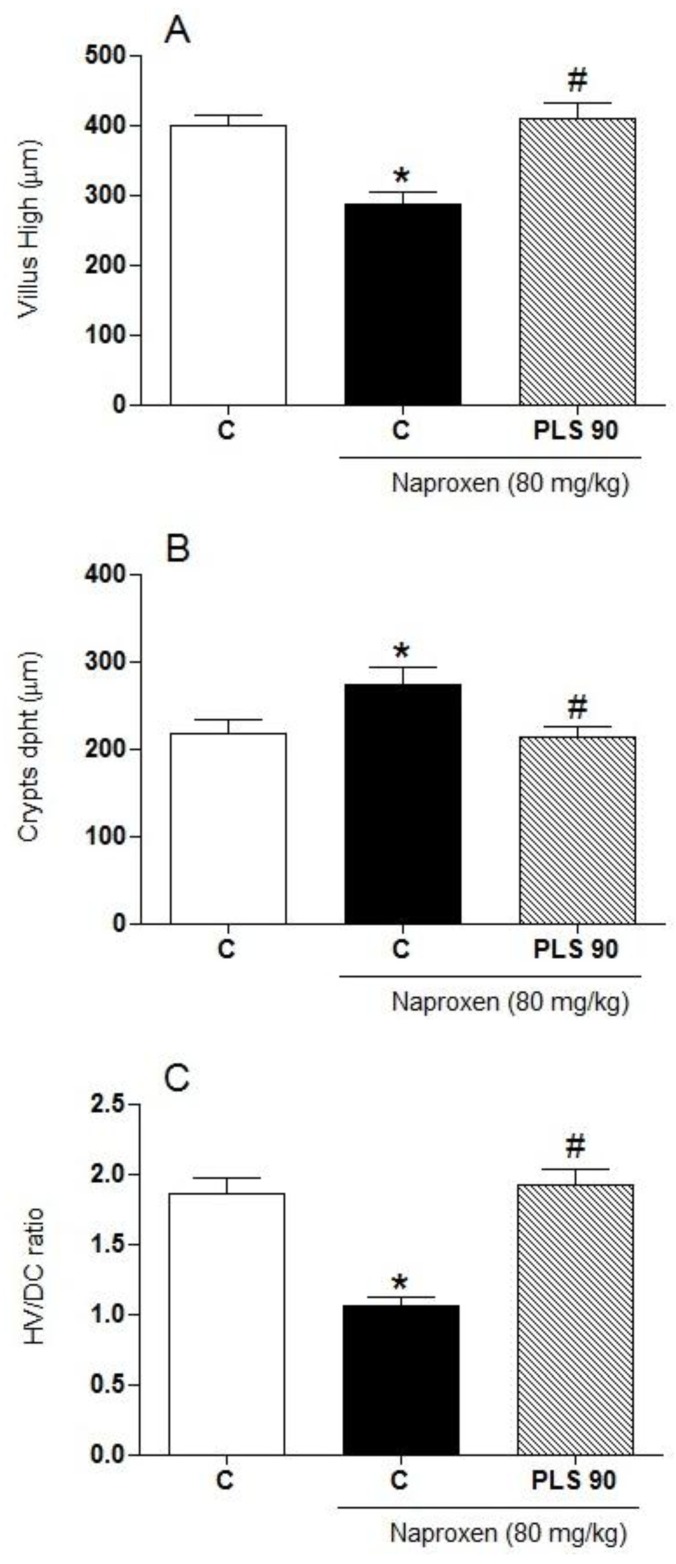
Morphometric analyses of the small intestine tissues in rats (*N *= 6) treated with naproxen alone or naproxen + the sulfated-polysaccharide fraction (PLS) extracted from *Gracilaria birdiae*. Rats were treated by gavage with either carboxymethylcellulose (C: control) or PLS (90 mg/kg) twice daily (at 09:00 and 21:00) for two days. After 1 h, naproxen (80 mg/kg) was administered by gavage. After 4 h, the animals were killed and segments of the small intestine were collected for the measurement of villus height (**A**), crypt depth (**B**), and the villus/crypt (VH/CD) ratio (**C**). * *p *< 0.05 *vs. *control group; # *p *< 0.05 *vs. *naproxen group (ANOVA and Newman-Keuls test).

Figure 2 shows that in the small intestine of the animals treated with naproxen (90 mg/kg, *p.o.*) twice daily for two days, significant villi shortening (Figure 2A), increased crypt depth (Figure 2B), and a decreased villus/crypt ratio (Figure 2C) were seen. However, when the animals were pretreated with PLS before naproxen administration, we observed a complete reversal of the intestinal morphometry alterations in the small intestine (Figure 2A,B,C). Other studies have shown that the development of NSAID-induced small intestinal ulcers is a multifactorial process, with a distinct pathogenesis of gastric damage [25] that involves a combination of events, such as increased epithelial permeability [26], intestinal hypermotility [27], and luminal bacterial invasion of the gut wall [28]. These effects lead to mucosal inflammation and eventually result in macroscopic damage. Taken together, these results indicate that PLS has a significant protective effect against naproxen-induced gastrointestinal damage.

**Figure 3:**
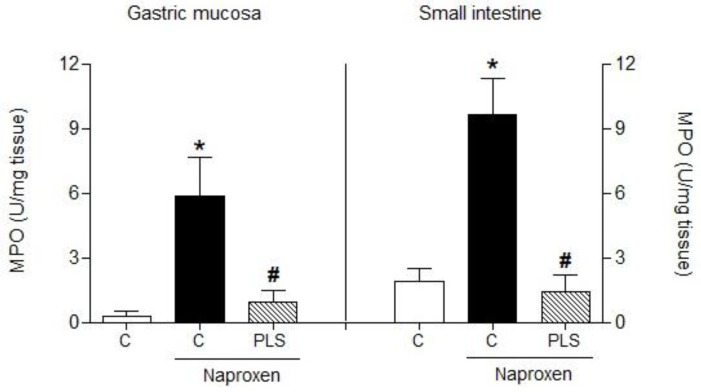
Effect of the sulfated-polysaccharide (PLS) fraction extracted from *Gracilaria birdiae *on gastric myeloperoxidase (MPO) activity in a rat model of naproxen-induced gastrointestinal damage. Rats were treated by gavage with either carboxymethylcellulose (C: control) or PLS (90 mg/kg) twice daily (at 09:00 and 21:00) for 2 days. After 1 h, naproxen (80 mg/kg) was administered by gavage. The results are expressed as the mean ± SEM of 5–7 animals per group. * *p *< 0.05 *vs. *control group; # *p *< 0.05 *vs. *naproxen group (ANOVA and Newman-Keuls test).

In recent years, studies have shown that PLSs interfere with cell migration at the site of inflammation. Researchers have shown that these compounds, including those isolated from algae, possess immunomodulatory activities that may stimulate the immune response, and thus control the anti-inflammatory response [29]. In this study, the accumulation of leukocytes following the appearance of naproxen-induced lesions was evaluated by measuring the activity of gastric myeloperoxidase (MPO). MPO exists in polymorphonuclear leukocytes (PNL) that produce an excessive amount of superoxide anions (O_2_^−^) and hydroxyl radicals (OH^•^), which are free oxygen radicals that are extremely toxic to the mucosa [30]. MPO levels have been shown to increase concomitantly with NSAID-induced injury in rats [31]. Furthermore, this observation is consistent with evidence indicating that mucosal damage induced by NSAIDs, such as naproxen, results in a marked increase in mucosal MPO activity, which may be associated with an increase in neutrophil infiltration and H_2_O_2_ in damaged tissues [32,33]. In this study, we observed significantly increased levels of MPO in the stomach (5.9 ± 1.7 U/mg of tissue) and the small intestine (9.7 ± 1.6 U/mg of tissue) of naproxen-treated rats compared to the control group (Figure 3). However, pretreatment with 90 mg/kg PLS significantly attenuated the naproxen-induced increase in MPO activity, in both the stomach (0.9 ± 0.5 U/mg of tissue) and the intestine (1.4 ± 0.7 U/mg of tissue). Therefore, PLS may protect the gastrointestinal tract by reducing the recruitment of leukocytes, thereby hindering superoxide anion production. 

Several studies have shown that NSAIDs also act as pro-oxidants, blocking the antioxidant systems of mucosal cells and resulting in the formation of reactive oxygen species (ROS). As a consequence of this process, oxidative damage occurs [34]. Recent works have demonstrated that ROS, such as superoxide anion and hydroxyl radicals, play an important role in the pathogenesis of various diseases, including the mucosal gastrointestinal damage induced by NSAIDs [35], ethanol [36], and others agents [37]. However, organisms have enzymatic and non-enzymatic defense mechanisms against the toxicity and tissue damage induced by these agents [38]. 

**Figure 4:**
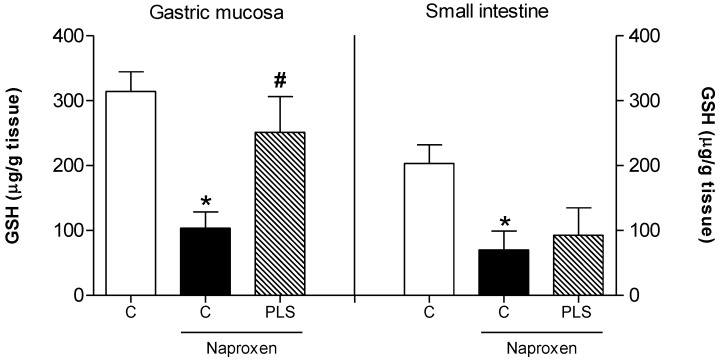
Effect of the sulfated-polysaccharide fraction (PLS) extracted from *Gracilaria birdiae *on glutathione (GSH) levels in rats with naproxen-induced gastrointestinal damage. Animals were treated by gavage with either carboxymethylcellulose (C: control) or PLS (90 mg/kg) twice daily (at 9:00 and 21:00) for 2 days. After 1 h, naproxen (80 mg/kg) was administered by gavage. The results are expressed as the mean ± SEM of 5–7 animals per group. * *p *< 0.05 *vs. *control group; # *p *< 0.05 *vs. *naproxen group (ANOVA and Newman-Keuls test).

**Figure 5:**
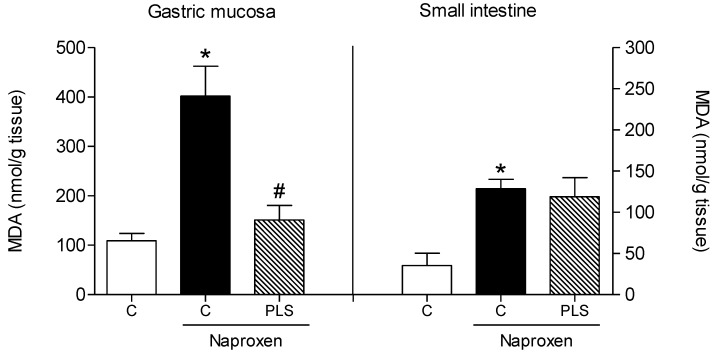
Effect of the sulfated polysaccharide (PLS) fraction extracted from *Gracilaria birdiae *on malondialdehyde (MDA) concentration in rats with naproxen-inducedgastrointestinal damage. Animals were treated by gavage with either carboxymethylcellulose (C: control) or PLS (90 mg/kg) twice daily (at 09:00 and 21:00) for 2 days. After 1 h, naproxen (80 mg/kg) was administered by gavage. The results are expressed as the mean ± SEM of 5–7 animals per group. * *p *< 0.05 *vs. *control group; # *p *< 0.05 *vs. *naproxen group (ANOVA and Newman-Keuls test).

Therefore, the present study investigated two oxidative stress markers, glutathione (GSH) and malondialdehyde (MDA). Naproxen significantly reduced the levels of GSH (103.4 ± 24.9 mg/g of tissue) and increased the concentration of gastric mucosal MDA (401.7 ± 61.5 nmol/g of tissue) in the stomachs of rats exposed to the drug compared to untreated normal controls (Figure 4 and Figure 5, respectively). Pretreatment with PLS (90 mg/kg) inhibited these effects in all of the naproxen-treated rats, reducing the naproxen effects and resulting in values that approximated those of controls without naproxen (251.1 ± 54.9 mg/g of tissue and 151.0 ± 29.8 nmol/g of tissue, respectively; Figure 4). These results suggested that the gastroprotective effect of PLS involves the participation of oxidative stress and its blockade. However, this alteration was not observed in the small intestine. Compared to the naproxen group (69.8 ± 29.3 mg/g of tissue and 214.2 ± 19.5 nmol/g of tissue, respectively), the group pretreated with PLS (90 mg/kg) did not show significant inhibition of GSH level depletion (92.5 ± 42.2 mg/g of tissue) or reduction in the concentration of MDA (198.1 ± 38.6 nmol/g of tissue) in the small intestine (Figure 4 and Figure 5).

Antioxidant compounds play an important role in various pathological conditions, including inflammation, neurodegenerative diseases, and cancer [39,40]. It has been systematically reported in literature that PLSs that show antioxidant activity, such as those extracted from marine algae, protect against cell death due to their ability to degrade excessive ROS [5]. However, few studies have correlated antioxidant potential with NSAID-associated injury of the gastrointestinal tract.

We have demonstrated that PLS significantly reversed the depletion of GSH levels in the gastric mucosa of rats treated with naproxen. These data are consistent with those reported in other studies demonstrating that PLSs from algae inhibited ethanol-induced gastric damage by increasing tissue GSH levels through the inactivation of ROS and products of lipid peroxidation [12]. Previous research suggested that ROS-induced damage can be prevented in two ways: by suppressing free-radical generation or by scavenging the free radicals generated [41]. Thus, we suggest that PLS prevents naproxen-induced gastropathy by increasing GSH levels and that this effect may be secondary to a decrease in free radical production. In addition, our results demonstrated that the administration of PLS results in a significant decrease in the concentration of MDA in naproxen-induced gastropathy. Thus, the mechanism through which PLS exerts its gastroprotective effect appears to involve a reduction in the lipid peroxidation induced by naproxen administration.

Concomitantly, our results showed that naproxen also decreases GSH levels and increases lipid peroxidation in the small intestine. Treatment with PLS did not inhibit the effects of naproxen on these biochemical parameters in this organ. Thus, we suggest that the mechanisms underlying the gastroprotective effect of PLS promote an increase in gastric mucosal resistance and a decrease in aggressive factors. In contrast to the gastric tissue, the increase in endogenous GSH levels in the small intestine does not appear to contribute to this effect. A possible mechanism may involve the downregulation of the inflammatory response by inhibiting the synthesis and release of pro-inflammatory mediators.

## 3. Experimental Section

### 3.1. Extraction of the Sulfated Polysaccharide Fraction

The marine alga *G. birdiae *was collected from the Atlantic coast of Brazil (Flecheiras Beach, Trairí, Ceará). The samples were cleaned of epiphytes, washed with distilled water, and stored at −20 °C. The polysaccharide extraction procedure was performed according to the method described by Farias *et al*. [42]. The dried tissue (5 g) was milled and suspended in 250 mL of 0.1 M sodium acetate buffer (pH 6.0) containing 510 mg of papain (E. Merck), 5 mM EDTA, and 5 mM cysteine, and incubated at 60 °C for 12 h. The residue was removed by filtration and centrifugation (2700× *g* for 25 min at 4 °C), and the sulfated polysaccharides were precipitated by the addition of 48 mL of 10% cetylpyridinium chloride (CPC, Sigma Chemical). The mixture was centrifuged (2700× *g* for 25 min at 4 °C) and the polysaccharides (kappa-carrageenan) in the pellet were washed with 200 mL of 0.05% cetylpyridinium chloride solution, dissolved in 174 mL of a 2 M NaCl/ethanol (100:15, v/v) solution, and precipitated with 200 mL of 70% ethanol (v/v) for 12 h at 4 °C. After further centrifugation (2700× *g* for 25 min at 4 °C), the precipitate was washed twice with 200 mL of absolute ethanol and dried with acetone under hot air flow (60 °C).

### 3.2. Chemical Characterization of the Sulfated Polysaccharide Fraction

The chemical characterization was previously determined [11,20]. Total sugar content of each fraction was determined according to the method of Dubois [43]. Protein content was measured by Bradford’s method [44]. Sulfate content in polysaccharides was determined by the barium chlorideegelatin method [45] and the monosaccharide composition of red seaweed galactans was obtained by reductive hydrolysis [46].

The peak molar masses (Mpk) were estimated by gel permeationchromatography (GPC) with a Shimadzu equipment at room temperature using an Ultrahydrogel linear column (7.8 × 300 mm, exclusion limits 106 g/mol), flow 0.5 mL/min, 0.5% polysaccharide concentration and 0.1 M NaNO_3_ as solvent.

### 3.3. Animals

Male Wistar albino rats, weighing 100–150 g, were obtained from the Department of Physiology and Pharmacology, Federal University of Ceará. All animals were housed in temperature-controlled rooms and received water and food *ad libitum*. The animals were deprived of food for 18–24 h before the experimentation, but had free access to water. All surgical procedures and animal treatments were conducted in accordance with the *Guide for Care and Use of Laboratory Animals *(National Institutes of Health, Bethesda, MD) and were approved by the local ethics committee (Protocol N^o^. 0066/10).

### 3.4. Effect of PLS on Naproxen-Induced Gastric Damage

Rats were pretreated with 0.5% carboxymethylcellulose (control group) or polysaccharide (PLS: 10, 30, and 90 mg/kg, *p.o.*) twice daily (at 09:00 and 21:00) for two days. After 1 h, naproxen (80 mg/kg, *p.o.*) was administered twice daily (at 09:00 and 21:00) for two days as described by Kim *et al.*, with modifications [17]. The control group received only vehicle or vehicle + naproxen. The rats were killed on the second day, 4 h after the naproxen treatment. The stomachs were promptly excised, opened along the greater curvature, and washed with 0.9% saline. The gastric damage was measured using digital calipers (Mitutoyo^®^). Samples of the stomachs were fixed in 10% formalin immediately after removal for subsequent histological evaluation. Others samples were then weighed, frozen, and stored at −70 °C until they were assayed for GSH levels [47], MDA concentration [48], and MPO activity [49]. 

### 3.5. Effect of PLS on Naproxen-Induced Small Intestine Damage

Animals were treated with 0.5% carboxymethylcellulose (vehicle) or PLS (90 mg/kg, *p.o.*) twice daily (at 09:00 and 21:00) for two days. One hour after the administration of PLS or vehicle, the animals were treated with naproxen (80 mg/kg, *p.o.*), as described above. The control group received vehicle or vehicle + naproxen. The rats were killed on the second day, 4 h after the naproxen treatment. The abdomens were then opened, and after identification of the intestine, a 5-cm portion of the small intestine (jejunum and ileum) was removed, washed with 0.9% saline, and pinned onto a wax block for the evaluation of macroscopic scores by modifying the criteria described by Morris *et al.* [50] (Table 2). All scoring of damage was performed in a randomized manner by an observer who was unaware of the treatments that the rats had received. Samples of tissue were then removed for the measurement of GSH levels [47], MDA concentration [48], and MPO activity [49]. Other samples were fixed in 10% formalin for subsequent histopathological and morphometric analysis. 

**Table 2:** Criteria for macroscopic scoring of intestinal damage.

Scores	Criteria
**0**	No damage.
**1**	Focal hyperemia; no ulcers.
**2**	Ulceration without hyperemia or bowel wall thickening.
**3**	Ulceration with inflammation at 1 site.
**4**	≥2 Sites of ulceration and inflammation.
**5**	≥2 Major sites of ulceration and inflammation or 1 site of ulceration extending >1 cm along length of intestinal.
**6–10**	If damage covered >2 cm along length of intestinal, score is increased by 1 for each additional cm of involvement.

### 3.6. Histological Evaluation of Gastric Damage

For histological evaluation, the stomach samples were fixed in 10% formalin solution for 24 h. After fixation, the samples were transferred to a solution of 70% alcohol. The material was then embedded in paraffin and sectioned; 4-µm-thick sections were deparaffinized, stained with hematoxylin and eosin (H & E), and then examined under a light microscope by an experienced pathologist without knowledge of the treatments. The specimens were assessed according to the criteria described by Laine and Weinstein, in which scores are assigned to the following parameters for a maximum total score of 14: hemorrhagic damage (score of 0–4), edema in the upper mucosa (score of 0–4), epithelial cell loss (score of 0–3), and presence of inflammatory cells (score of 0–3) [51].

### 3.7. Intestinal Morphometric Analysis

Morphometric analysis was performed using slides stained with H & E on a light microscope equipped with a high-resolution Leica DFC 320 digital camera (Wetzlar, Germany) connected to a computer with an image capture program. An average of 8 to 10 different linear measurements of crypt depth and villus height were recorded. The height of the villus was measured from the top to the bottom, corresponding to the junction of the crypt/villus. The depth of the crypts was defined as the invagination between adjacent villi.

### 3.8. Glutathione Levels

The concentration of glutathione (GSH) in the samples of stomach and small intestine tissues was estimated according to the method described by Sedlak and Lindsay [47]. A segment from each organ was homogenized in 5 mL of cold 0.02 M EDTA solution (1 mL 100 mg/tissue). Aliquots (400 μL) of the tissue homogenate were mixed with 320 μL of distilled water and 80 μL of 50% (*w/v*) trichloroacetic acid in glass tubes and centrifuged at 3000 rpm for 15 min. Next, 400 μL of each supernatant was mixed with 800 μL of Tris buffer (0.4 M, pH 8.9) and 20 μL of 0.01 M 5,5-dithio-bis (2-nitrobenzoic acid). Subsequently, the samples were stirred for 3 min and read on a spectrophotometer at 412 nm. GSH concentration was determined via a reduced GSH standard curve, which was generated in parallel. The results are expressed as micrograms of GSH per gram of tissue.

### 3.9. Malondialdehyde Concentration

The MDA levels in the homogenate from each group were measured using the method described by Mihara and Uchiyama [48], which is based on the reaction with thiobarbituric acid. Fragments of the gastric mucosa and small intestine weighing between 100 and 150 mg were homogenized with cold 1.15% KCl to prepare 10% homogenates. Briefly, 250 μL of each homogenate was added to 1.5 mL of 1% phosphoric acid (H_3_PO_4_) and 0.5 mL of 0.6% *tert*-butyl alcohol (aqueous solution). Then, this mixture was stirred and heated in a boiling water bath for 45 min. The mixture was then cooled immediately in an ice water bath followed by the addition of 4 mL of *n*-butanol. This mixture was shaken and the butanol layer was separated by centrifugation at 1200× *g* for 10 min. Optical density was determined to be 535 and 520 nm, and the optical density difference between the two determinations was calculated as the *tert*-butyl alcohol value. MDA concentrations are expressed as millimoles per gram of tissue.

### 3.10. Myeloperoxidase Activity

Myeloperoxidase is an enzyme found primarily in neutrophil azurophilic granules which has been used extensively as a biochemical marker for granulocyte infiltration into various tissues, including the gastrointestinal tract. The extent of neutrophil accumulation in the gastric mucosa and small intestinal was measured by MPO activity evaluation [49]. Briefly, 50–100 mg of tissue was homogenized in 1 mL of potassium buffer with 0.5% of hexadecitrimetilamônio (HTAB) for each 50 mg of tissue. Then, homogenate was centrifuged at 40.000× g for 7 min at 4 °C. MPO activity in the resuspended pellet was assayed by measuring the change in absorbance at 450 nm using o-dianisidine dihydrochloride and 1% hydrogen peroxide. The results were reported as the MPO units per mg of tissue. A unit of MPO activity was defined as that converting 1 µmol of hydrogen peroxide to water in 1 min at 22 °C.

### 3.11. Statistical Analysis

Data were described as either means ± SEM or median, as appropriate. Analysis of Variance (ANOVA) followed by Student-Newman-Keuls test was used to compare means and Kruskal-Wallis nonparametric test, followed by Dunns tests to compare medians; *P* < 0.05 was defined as statistically significant.

## 4. Conclusions

Our results suggest that PLS has a beneficial effect in this model of naproxen-induced gastrointestinal damage, through mechanisms that involve the inhibition of inflammatory cell infiltration and lipid peroxidation. We suggest that polysaccharides may have potential applications in the development of novel therapeutics for preventing NSAID-induced adverse effects in the gastrointestinal tract in humans.
